# Roles of dendritic epidermal T cells in steady and different pathological states

**DOI:** 10.1093/burnst/tkae056

**Published:** 2025-02-12

**Authors:** Jiaqi Hao, Jie Zhang, Yan Liu

**Affiliations:** Department of Burn, Shanghai Burn Institute, Ruijin Hospital Affiliated to Shanghai Jiao Tong University School of Medicine, 197 Ruijin 2nd Road, Huangpu District, Shanghai 200025, China; Department of Burn, Shanghai Burn Institute, Ruijin Hospital Affiliated to Shanghai Jiao Tong University School of Medicine, 197 Ruijin 2nd Road, Huangpu District, Shanghai 200025, China; Department of Burn, Shanghai Burn Institute, Ruijin Hospital Affiliated to Shanghai Jiao Tong University School of Medicine, 197 Ruijin 2nd Road, Huangpu District, Shanghai 200025, China

**Keywords:** Dendritic epidermal T cells, Wound healing, Skin immune surveillance, Re-epithelialization, Keratinocytes

## Abstract

The epidermis is the outermost layer of the skin and acts as the primary barrier to protect the body. Dendritic epidermal T cells (DETCs), which are specifically distributed in epidermal tissues, play a crucial role in skin immune surveillance and wound healing. DETCs are one of the most important components of the epidermis and exert a steady-state monitoring function, facilitating wound healing and tissue regeneration after skin injury. Skin wounds are often linked to other pathological conditions such as ageing, ultraviolet radiation, and metabolic diseases such as diabetes mellitus and obesity. Therefore, it is crucial to investigate how DETCs regulate themselves and the external environment during these pathological states. DETCs interact closely with keratinocytes in the epidermis, and this intercellular interaction may be essential for maintaining health and integrity. In this review, we focus on the characteristics and underlying mechanisms of DETCs in maintaining epidermal homeostasis and re-epithelialization in different pathological states.

Highlights

- DETCs are a specific type of T cell in the epidermis and play a crucial role in epidermal homeostasis maintenance and wound healing.
- DETCs interact closely with keratinocytes in the epidermis and regulate the external environment during pathological states.
- The roles of DETCs in pathologic conditions are elucidated.
- Recent technical advances in DETCs research are briefly introduced.

## Background

Although both types of T cells develop in the thymus, they can be classified into two groups based on their T cell receptors (TCR): αβT cells, which express αβTCRs, and γδT cells, which have TCRs comprising TCR-γ and TCR-δ chains [1,2]. Although αβT cells are typically found in secondary lymphoid organs, γδT cells, which serve as the first line of defence, are primarily located in epithelial tissues, such as the skin, reproductive tract, gastrointestinal tract, and respiratory tract [3].

In mice, γδ T cells are divided into seven subsets (from Vγ1 to Vγ7 T cells) based on their distinct TCR γ chains. Among these subsets, dendritic epidermal T cells (DETCs) are the only T cells that express an invariant Vγ5Vδ1 TCR and reside exclusively in the murine epidermis. DETCs possess dendritic morphology and comprise >90% of murine T lymphocytes [4].

Similarly, human γδT cells can be subdivided into Vδ1 and Vδ2 based on the two variable regions of TCR-δ [5]. While γδ2+ T cells dominate peripheral blood, γδ1+ T cells are primarily expressed in the dermis and epidermis of human skin samples [5]. This suggests that in epithelial tissues, γδ + T cells primarily express the γδ1 chain [6–9]. Furthermore, unlike rodents, human skin contains both αβT and γδT cells at a ratio of ~5 : 1 [6,10].

Although an exact homologue of murine DETCs has not been identified in humans [11], human epidermal Vγδ1+ T cells have been shown to share similar biological functions with murine DETCs, secreting growth factor including insulin-like growth factor I (IGF-1) [10], keratinocyte growth factor-1/2 (KGF-1/2), transforming growth factor-β (TGF-β), granulocyte-macrophage colony-stimulating factor (GM-CSF), and interferon-γ (IFN-γ) [12].

Therefore, although human skin Vγδ1+ T cells do not possess the same dendritic morphology and distribution as murine DETCs [10,13], studying murine DETCs will contribute to a better understanding of the role of T cells in human skin.

As the only lymphocytes residing in the epidermis, DETCs function as the first line of defence and are in close contact with other epidermal cells, such as keratinocytes and Langerhans cells. DETCs have immune surveillance, antitumour, and epithelial growth-promoting properties. They are of great physiological and clinical significance and have gained increasing research focus.

This study reviews the biological characteristics of DETCs in mice and their roles in maintaining epidermal homeostasis and regulating wound healing in both physiological and pathological states.

## Review

### DETCs in homeostasis maintenance

DETC activation requires two signals: a pre-activation signal generated by TCR and a co-stimulatory signal from co-receptors, including junctional adhesion molecule-like (JAML), natural killer group 2 member D (NKG2D), and CD100. Unlike classical T cells, DETCs remain in a state of incomplete activation until they receive a co-stimulatory signal.

**Figure 1:**
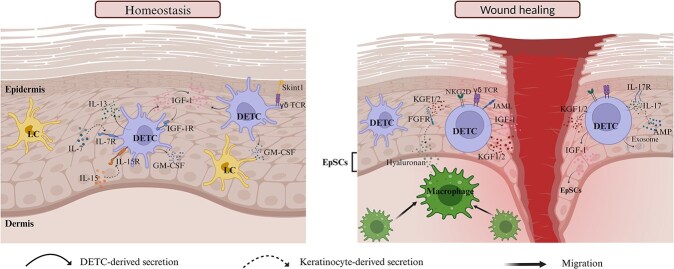
Role of DETCs in homeostasis and wound healing. In the steady state of the skin, DETCs constitutively produce cytokines, including IGF-1 and IL-13, to regulate keratinocyte proliferation and apoptosis, the latter of which also mediates homeostatic maintenance. DETCs are the main source of GM-CSF, which is crucial for the maturation of Langerhans cells. Cytokines from the epidermal cells contribute to DETC homeostasis. Skint1–γδTCR interaction plays an essential role in maintaining epidermal integrity and promoting immunosurveillance of DETCs. This molecular bridge integrates both innate and adaptive immunities. DETCs are completely activated upon skin injury. DETCs and DETC-derived cytokines promote wound repair by regulating keratinocytes, epidermal stem cells, and macrophages. *γδTCR* γδT cell receptor, *LC* langerhans cells, *DETC* dendritic epidermal T cell, *IL-13* interleukin-13, *IL-7* interleukin-7, *IL-7R* interleukin-7 receptor, *IL-15* interleukin-15, *IL-15R* interleukin-15 receptor, *GM-CSF* granulocyte-macrophage colony-stimulating factor, *Skint1* selection and upkeep of intraepithelial T cells 1, *EpSCs* epidermal stem cells, *KGF-1/2* keratinocyte growth factor-1/2, *IGF-1* insulin-like growth factor 1, *JAML* junctional adhesion molecule-like, *IL-17* interleukin-17, *IL-17R* interleukin-17 receptor, *AMP* antimicrobial peptides, *FGFR* fibroblast growth factor receptor, *NKG2D* natural killer group 2 member D. Created with BioRender.com

In the steady state of the skin, DETCs are typically semi-activated and maintain permanent contact with epidermal cells through their unique dendritic structures. This allows them to perform immune surveillance of the epidermis. DETCs secrete IGF-1, interleukin (IL)-13, and other molecules that support keratinocyte proliferation and growth. Furthermore, factors secreted from other epidermal cells regulate homeostasis and the physiological state. Interactions between DETCs and other epidermal cells are crucial for maintaining skin homeostasis (Figure 1).

#### Pre-activated state of DETCs through TCRs

DETCs are semi-activated or pre-activated, even in the absence of infection or tissue wounding [14]. This is because of the unique γδT TCRs.

DETCs are located in the epidermis and have a well-characterized dendritic morphology. Most dendrites are anchored to the apical epidermis and immobilized at the distal end, whereas the remaining dendrites project to the basal epidermis, extending and contracting in a highly mobile state [15]. Prominent co-clusters of TCRs and proteins phosphorylated on tyrosine (pY) were detected at the ends of apically oriented dendrites using intravital dynamics–immunosignal correlative microscopy. These pY-containing structures are co-localized with apical dendrite anchoring sites and are termed immunological synapse-like phosphotyrosine-rich aggregates located on projections [15,16].

The formation of phosphotyrosine-rich aggregates located on projections depends on Lck, which mediates the activation of the Vγ5 TCR. This further maintains DETCs in a state of steady activation, whereas epithelial integrin αE(CD103)β7 helps anchor their long cellular projections towards the apical epidermis [14,15]. Due to this specific structure, DETCs gather mature lysosomes at the barrier-forming apical epidermis through long-range trans-epithelial transport, probing the molecular composition of the epidermis [14,15], so as to receive continuous low-level TCR signals from adjacent cells residing in the epidermis and maintain the semi-activated state of DETCs in healthy skin. This activatory interaction, resembling an immunological synapse, enhances T cell surveillance at tight junctions and may contribute to intercellular communication and ligand pattern-based stress detection.

In TCRδ^−/−^ mice, αβ T cells, acting as substitutes for γδT cells, are present in the epidermis and can be activated by direct stimulation [17]. However, DETCs expressing αβ TCRs are not retained throughout an animal’s life and cannot recognise damaged keratinocytes during wound repair. This indicates that keratinocyte-responsive TCR is essential for DETC maintenance in the epidermis and its activation in response to damaged keratinocytes [17].

Depletion of the linker for the activation of T cells, an essential signalling molecule downstream of TCR, impairs TCR signalling and DETC activation, leading to delayed wound healing [18].

However, ligands binding to DETC TCR have not yet been characterized. Komori *et al.* used soluble DETC-TCR tetramers and found that DETC tetramer binding was only observed in keratinocytes near the wound edge. In contrast, those located distal to the wound site did not bind to DETC tetramers [19], suggesting that ligands of DETC TCR are not detectable in healthy skin. Low expression level of TCR ligands in the steady state may explain this controversial result, as current detection methods may not be sufficiently sensitive. Further investigations are needed to understand the natural ligands of TCRs and the regulatory mechanisms that maintain DETCs. In addition, when identifying TCR ligands, it is important to prioritize physiologically expressed molecules over stress-upregulated molecules.

#### Interaction between DETCs and the epidermis

In the steady state, semi-activated DETCs express CD69 and CD122 on their cell surfaces, which can be seen as activation markers. They also produce several cytokines, including IGF-1, IL-13, and GM-CSF, which function in skin homeostasis.

DETCs constitutively produce low-dose IGF-1, which acts on keratinocytes to increase proliferation, reduce apoptosis, and maintain keratinocyte homeostasis [20]. DETCs also express IGF-1 receptor (IGF-1R), indicating that IGF-1 can reduce DETC apoptosis, forming an autocrine feedback loop that mediates homeostatic maintenance of DETC numbers in the skin [20]. Furthermore, DETCs constitutively produce IL-13, a barrier maintenance cytokine vital for maintaining skin homeostasis and restoring epithelial tissue integrity following insult [21]. IL-13 contributes to protection against cutaneous epithelial carcinogenesis and controls the rate of epithelial cell movement from the basal layer, promoting the upward maturation of skin epithelial cells and epithelial renewal. Mice lacking IL-13 showed enhanced transepidermal water loss and defects in the restoration of epidermal integrity [21]. The IL-13-producing DETCs in the adult epidermis differentiate from their fetal thymic precursors, which are preprogrammed to produce IFN-γ. Latest research found that this functional differentiation of DETCs depended on the TCR/p38 mitogen-activated protein kinase (MAPK) signalling pathway and was accompanied by a metabolic transition where glycolytic capacity in adult DETCs in steady state was lower than that in proliferating neonatal DETCs [22]. When stimulated, adult DETCs induced high glycolytic capacity and IFN-γ production during the late phase of activation, whereas inhibition of glycolysis could dampen production of IFN-γ but not IL-13 [22]. This suggested that cellular metabolic states of DETCs may fine-tune their effector functions. Whether cellular metabolism has a role in regulating other functions of DETCs, including cytotoxicity and cytokine production, is worthy of further study.

DETCs are also the primary source of GM-CSF [23], which is crucial for the maturation of Langerhans cells [23]. In mice with a DETC-deficient epidermis, low GM-CSF levels are associated with impaired Langerhans cell maturation [24]. This finding is consistent with the finding that DETCs are the main source of GM-CSF in the epidermis. In addition, when cultured with Langerhans cells, the expression of gamma c receptors on DETCs is elevated, and DETCs acquire proliferative responsiveness to their own growth factor, IL-15 [23]. This suggests an interaction between DETCs and Langerhans cells, during which they regulate the residence and function of each other.

At a steady state, DETCs also express granzyme C [25,26], which responds to both environmental stimuli and TCR engagement. The unique localization of DETCs in the epidermis and the distribution of granzyme C along dendritic extensions suggest that DETCs distribute granzyme C widely in the epidermis, possibly reacting quickly to viral infections [26].

DETCs play a role in immune surveillance by exerting defensive functions against virus-infected, transformed, and stressed cells. Cytokines in epidermal cells contribute to DETC homeostasis.

IL-7 is crucial for γδT cell development and is secreted by keratinocytes, fibroblasts, mesenchymal cells, and thymic epithelial cells. IL-7 exerts its biological role by interacting with IL-7R, a heterodimer composed of an α-chain and a γc-chain. This interaction activates the JAK-signal transducer and activator of transcription (STAT) and phosphatidylinositol 3-kinase (PI3K)/Akt protein kinase B pathways. IL-7 plays a crucial role in the thymic differentiation of Vγ3 cells. In mice lacking IL-7 or IL-7Rα, the maturation of fetal Vγ3 thymocytes was almost blocked. The interaction between IL-7 and IL-7R, via activated-Stat5, promotes recombination and transcription at the IgH and TCRγ loci by binding to consensus motifs in the 5′ regions of the Jgamma segments [27]. Il-7Rα-deficient mice exhibit severe impairment in V–J recombination of the T cell receptor-γ gene [28,29]. However, the constitutively active form of Stat5 partially restored it, thereby rescuing T cell development from IL-7R^−/−^ γδ T precursors [27]. Furthermore, IL-7 promotes the expression of the CD3/TCR phenotype in DETCs [30], suggesting that IL-7 plays a role in phenotypic maturation. Keratinocyte-derived IL-7 contributes to the survival and growth of DETCs, which can be enhanced by TNF-α [31,32]. The IL-7-mediated communication between epithelial cells and γδ T cells plays a crucial role in maintaining homeostasis.

IL-15, a cytokine produced in both lymphoid and non-lymphoid tissues, promotes T cell proliferation and differentiation. Keratinocytes are a major source of IL-15 [33]. The IL-15 heterotrimer receptor shares two chains (β and γc) with the IL-2 receptor, indicating similar biological functions. However, IL-15Rα (CD215) is expressed more on DETCs than IL-2Rα (CD25), suggesting a greater impact of IL-15 than IL-2 [33]. IL-15 is crucial for the phenotypic maturation of DETCs in the fetal thymus. IL-15 signalling via IL-2Rβ promotes selective localiztion of DETCs in the epidermis of adult mice. Furthermore, IL-15 facilitated IGF-1 production by DETCs via the mammalian target of rapamycin (mTOR) pathway [34]. Moreover, IL-15 enhances granzyme C expression in DETCs, contributing to antiviral immunity in tissues [26]. IL-15, along with IL-7, helps maintain DETC homeostasis.

Semi-activated DETCs are in constant contact with neighbouring keratinocytes and Langerhans cells through the molecular bridges mentioned above, integrating innate and adaptive immunity.

#### Skint 1

FVB/N mice from Taconic Farms (FVB/N Tac) lack Vγ5Vδ1 DETCs in the epidermis, whereas their γδ T cell repertoires in other tissues remain normal [35]. This finding led to the identification of the selection and upkeep of intraepithelial T cell protein 1 (Skint1) [36], which is butyrophilin-related, in 2008. Since then, Skint1, a member of a novel immunoglobulin superfamily gene cluster encoding a 364 amino acid protein, has received much attention [36]. Skint1 is exclusively expressed in thymic epithelial cells and epidermal keratinocytes [37]. During development, Skint1, expressed on the surface of thymic epithelial cells, selects Vγ5 + Vδ1+ T cells in the thymus depending on the complementarity-determining region-like loop of immunoglobulins within the membrane distal variable domain of Skint1 [36], thus playing an important role in the development of Vγ5 + Vδ1+ thymocytes [38]. Mice harbouring a null mutation in Skint1 lack canonical Vγ5Vδ1-expressing DETCs [38].

Because Skint1 is specifically expressed in the thymus and epidermis, and Skint1 in the thymus has been shown to directly promote the functional differentiation of DETC progenitors, it is reasonable to suggest that Skint1 exerts a unique function in the epidermis, possibly in the selective localization and maintenance of Vγ5 + Vδ1+ T cells in the skin. In the steady state, the interaction between DETCs and keratinocytes requires constitutive DETC TCR-containing foci and the sustained expression of Skint1 in differentiated keratinocytes [39]. This Skint1–γδTCR interaction plays an essential role in maintaining epidermal integrity, as sustained Skint1 disruption results in decreased expression of DETCs and keratinocyte-derived barrier maintenance molecules and increased apoptosis of keratinocytes. Furthermore, steady-state Skint1 promotes immunosurveillance of DETCs by maintaining a predetermined effector phenotype [39].

Furthermore, it is worth mentioning that the butyrophilin family, to which Skints belong, plays either a co-inhibitory or co-stimulatory role in regulating T-cell function [40]. This suggests that Skint family members may also act as immune system regulators. Skint2, also known as B7S3, is also required for skin DETC development [41], although it cannot compensate for Skint1 in mediating DETC selection [37]. Additionally, it has also been found to inhibit CD8+ T-cell activation in mice [42].

Currently, various aspects of Skint1 remain unknown. For example, it is unclear how the DETC–Skint1 interaction recovers after stress resolution and how Skint1 expression is regulated by DETCs in keratinocytes. Further investigation is required to understand the crosstalk between TCR–Skint1 and its impact on DETC homoeostasis and the epithelial barrier.

### Role of DETCs in wound healing

Wound healing is a complex and dynamic biological process that can be divided into four stages: hemostasis, inflammation, proliferation, and tissue remodelling [43]. As DETCs are the only lymphocytes in the epidermis, their role in wound healing is of great importance. Upon skin injury, DETCs are rapidly activated by stressed keratinocytes via co-stimulatory receptors. Once fully activated, DETCs interact with other cells and are largely involved in the repair process (Figure 1).

Here, we review the exact mechanisms required for the full activation of DETCs, how DETCs regulate keratinocytes and epidermal stem cells for proper re-epithelialization, and how DETCs communicate with macrophages during wound healing.

#### Complete activation of DETCs in wound healing

While mature DETCs exist in a semi-activated state under homeostatic conditions, full activation of DETCs in response to skin injury is essential, comprising both pre-activation signals, as mentioned above, and co-stimulatory molecules. To date, three co-stimulatory molecules have been studied extensively.

JAML, 55 kDa, is an epithelial γδ T cell-specific co-stimulatory molecule. While low-level expression of JAML was found in resting γδ T cells isolated from mouse epidermis, 70–95% of tissue-derived γδ T cells upregulated JAML upon stimulation [44]. Ligation of JAML to coxsackie and adenovirus receptor (CAR) [45] induces proliferation and upregulates KGF-1 expression and IL-2 production in DETCs [44]. Disruption of the interaction between CAR and JAML results in the isolation of less-activated DETCs from the wound margin and impaired wound repair [44].

CD100, also known as axon-guided factor 4D, is another characteristic signalling protein in T cells [46]. The binding of CD100 to its ligand plexin B2 leads to rapid and transient phosphorylation of extracellular regulated protein kinases (ERK) and dephosphorylation of cofilin [46,47], inducing cellular rounding in DETCs [46], which is one of the first observable DETC responses to wounding. Consistently, in the absence of CD100-mediated signals, DETC activation was inhibited, and the conversion from dendritic to round shape was disrupted, resulting in the delayed repair of cutaneous wounds.

Of these three co-stimulatory molecules, NKG2D has been the best studied. NKG2D, a receptor for major histocompatibility complex (MHC) class I chain-related molecules A/B (MICA/B) and UL16 binding proteins in humans [48,49], is normally detected in γδ T cells, CD8+ αβT cells, and natural killer (NK) cells [49]. Unlike NK cells and CD8+ T cells, DETCs constitutively express both NKG2D isoforms, NKG2D-S and NKG2D-L, and two associated adaptor proteins, DAP10 and DAP12 [47]. Ligands that activate NKG2D receptors in the early stages of wound injury include retinoic acid early inducible-1 (RAE-1) [50,51], mouse UL16-binding protein-like transcript 1 (Mult1) [52,53], and histocompatibility 60-c (H60c).

Acute upregulation of RAE-1 in the epidermis, which often occurs during chemical carcinogenesis [54], has been shown to induce morphological and activation changes in both Langerhans cells and DETC compartments *in vivo* [51], initiating a rapid and diverse immune surveillance response *in vivo*.

H60c is a major NKG2D ligand expressed in keratinocytes at the wound margin [55], whereas RAE-1 and Mult-1 are not detected [56]. A significant increase in H60c mRNA levels was detected after injury [55]. Consistently, the H60c protein was not clearly detectable until 3 h after wounding, and its expression level increased between 6 and 12 h after wounding [56].

H60c/NKG2D interactions play a critical role in wound repair, as blockade of H60c/NKG2D leads to decreased KGF production and impaired wound healing [56].

NKG2D ligation has been shown to induce cytotoxicity and cytokine production in DETCs in the absence of TCR engagement [57], suggesting that NKG2D is a ligand that can be activated independently of TCR. Ibusuki *et al.* showed that DETC-mediated lysis of keratinocytes could be abolished using a PI3K inhibitor, suggesting that NKG2D depends on PI3K, but not on the Syk/ZAP70 signalling pathway, to directly induce the cytotoxicity of DETCs [58].

Recently, heat shock protein family A member 8 (Hspa8) and intercellular adhesion molecule-1 (ICAM-1), both expressed by keratinocytes, were identified as additional co-stimulatory molecules for DETCs [59]. After rapid upregulation in the epidermis upon injury, Hspa8 and ICAM-1 upregulated CD25 expression in DETCs, induced proliferation, and increased IL-2 production, thus playing a role in the DETC-mediated damage response. In the absence of Hspa8, the ability of keratinocytes to activate DETCs is reduced, and wound repair is significantly impaired [59].

DETCs are regulated not only by positive signalling but also by negative signalling. DETCs express inhibitory receptors such as Ly49E, CD94/NKG2, and programmed death-ligand 1 (PD-1). CD94/NKG2 ligation inhibits the cytolytic activity of DETCs [60]. Differing significantly from those isolated from acute wounds, skin γδT cells isolated from chronic wounds are resistant to further stimulation and are unresponsive. This may be attributed to the increased expression of inhibitory receptors, such as PD-1, which is elevated in response to severe injury, inhibiting proliferation and cytokine production [61,62].

E-Cadherin acts as an inhibitory receptor on DETCs, whereas αE(CD103)β7 integrin, another receptor for E-cadherin expressed on DETCs, acts as a co-stimulatory receptor. Downregulation of E-cadherin on activated DETCs, a known tumour suppressor protein, impedes DETC activation and effector molecule production [63]. In contrast, αE(CD103)β7 integrin on DETCs binds to E-cadherin on keratinocytes and forms short-term adhesions between DETCs and keratinocytes, allowing DETCs to lyse keratinocytes [63]. The exact signalling pathways that mediate this inhibitory signal through E-cadherin require further study.

CD200 receptor is another inhibitory receptor expressed in DETCs [64]. After activation, DETCs showed an increased expression of CD200 receptor. Ligation of CD200 receptor with CD200 resulted in inhibition of proliferation and reduced cytokine secretion. As a p53-target gene, CD200 is upregulated in murine dendritic cells during apoptosis, contributing to suppressed immune reactivity to ultraviolet (UV) B radiation-mediated self-antigens [65]. This suggests that CD200 signalling in DETCs may also partly participate in immune escape after UV radiation.

#### Regulation of keratinocytes by DETCs

Once fully activated, DETCs secrete large amounts of cell factors, such as IGF-1 and KGF, which regulate the proliferation and migration of keratinocytes and protect them from apoptosis, ultimately enhancing the wound healing process.

The primary source of IGF-1 in the epidermis, DETCs, constitutively produce low doses of IGF-1 at a steady state to maintain keratinocyte homeostasis [20]. Once activated, IGF-1 production increases, and it binds to the heterotetrameric IGF-1R, a member of the transmembrane tyrosine kinase receptor family [66,67]. Ligation of IGF-1 and IGF-1R leads to autophosphorylation of tyrosine residues in the cytoplasmic region of the receptor beta subunit, which induces phosphorylation of downstream signalling pathways, including the insulin receptor substrate proteins that act as docking proteins [68,69], and finally leads to the activation of the Ras/Raf/MAPK and PI3K/Akt/p70S6K pathways [70], which mediate several intracellular signals associated with cell proliferation and survival [67,68] and are involved in mediating the IGF-1R inhibitory signal on keratinocyte differentiation [71–73]. Besides the upregulated production of IGF-1, TCR stimulation triggers increased expression of the IGF-1R on DETCs, possibly forming a positive feedback loop to enhance the potency of DETCs during wound healing [20]. IGF-1 also induces the expression of c98, which has a high sequence identity with the Bcl-2 family, to prevent keratinocyte apoptosis [66,67]. The anti-apoptotic effects of IGF-1 through c98 have been speculated to be related to its bcl-2 homology domains 1, 2, and 3, and are more pronounced during the high mitotic stage of the skin epidermis [68,69].

IL-15 in the epidermis is not only essential for the homeostasis of DETCs but also promotes DETC activation and IGF-1 generation. In streptozotocin-induced diabetic mice, the reduction of epidermal IL-15 resulted in the impairment of DETC activation and IGF-1 production, whereas the application of exogenous IL-15 helped to restore them [74]. Wang *et al.* found that the addition of rIL-15 or recombinant insulin-like growth factor-1 (rIGF-1) to the wound bed increased epidermal IGF-1 or IL-15 production, suggesting a positive correlation between IL-15 and IGF-1 [74].

However, it has been shown that in chronic refractory wounds, epidermal T cells become less responsive to activation, functionally impaired, and unable to produce IGF-1 during the tissue repair process [10]. This dysfunction, which may be contributed to T cell unresponsiveness to signalling activation or increased expression of inhibitory receptors, remains elusive. One potential mechanism is that during wound repair, IL-1β activates nuclear factor kappa-B (NF-κB) signalling in DETCs, significantly increasing the expression of let-7f-5p, an IGF-1-specific miRNA, which ultimately inhibits the posttranscriptional expression of *IGF1* mRNA and delays wound healing [75].

In contrast to IGF-1, DETCs only express KGF-1/2 when stimulated, as KGF-1 mRNA was detected in DETCs isolated from the wound area but not from non-wounded skin [76,77].

Although DETCs secrete both KGF-1 and KGF-2 within 24 h of injury [78], KGF-1 was the first to be discovered. Despite numerous reports showing the importance of fibroblast growth factor (FGF)-7 in wound healing, a study by Guo *et al.* in 1995 found that FGF-7 was not a critical factor, as FGF-7–TGF-α double-knockout mice showed normal wound healing [79]. Similarly, other studies have shown that the absence of KGF-1 does not result in abnormal epidermal growth or impaired wound healing. This suggests that FGF-7 may be compensated for by other KGF-receptor ligands. FGF-10, which shares sequence homology and structural similarity with KGF-1, is a strong candidate for compensation [76,79–81]. It was later demonstrated that FGF-7 and FGF-10, which are expressed in the dermal compartment of the skin rather than the epidermal compartment [82], share a similar expression pattern. Moreover, FGF-10 binds to FGF receptor2-IIIb (FGFR2IIIb) with high affinity [76,83] and is even more effective than KGF-1 and TGF-β in wound closure and scar formation [84,85]. Phosphorylated KGF receptor triggers downstream signalling pathways, including PI3K/AKT, mTOR, and MAPK, leading to the morphogenesis, proliferation, and migration of keratinocytes when binding to the KGF receptor [86]. Recombinant KGF has been observed to restore wound healing in DETC-deficient skin [83,85,87], indicating the importance of DETCs in re-epithelialization through the production of KGF-1/2 upon stimulation.

### Regulation of epidermal stem cells by DETCs

Epidermal stem cells (EpSCs) are located in the basal layer of the epidermis and can migrate to injured areas of the skin [88]. When robustly activated, EpSCs and their progeny efficiently recruit toward epidermal lineage, significantly accelerating wound repair. This makes them promising candidates to treat skin wounds [89–91]. Recent studies have shown that DETCs affect EpSCs, accelerating wound healing. There was no significant difference in the proportion of EpSCs in the normal epidermis between wild-type mice and mice lacking DETCs. However, DETCs significantly increase the proportion of EpSCs in the epidermis surrounding the wound [92,93]. This is a potentially critical mechanism for the enhanced DETC-mediated re-epithelialization. DETCs secrete IGF-I to promote the proliferation of EpSCs while inhibiting their differentiation into end-stage cells. This improves the proliferation potential and anti-apoptotic ability of newly formed epidermal tissue, ultimately promoting wound healing [92]. Exosomes, which are secreted from host cells and taken up by target cells, act as vital messengers involved in multiple forms of intercellular communication [94]. Liu *et al.* used biomarkers, such as CD49f-CD71, K15, and BrdU, to identify variations in EpSCs. They found that DETCs promoted the proliferation of EpSCs by secreting exosomes. These exosomes were oval-shaped with a low-density middle area under transmission electron microscopy [93].

Further research is necessary to clarify the exact content of DETC-derived exosomes, as they are crucial signalling mediators for skin DETCs and have significant potential for promoting the proliferation of EpSCs for wound re-epithelialization.

#### Interaction between DETCs and macrophages

Recent studies have shown that macrophage polarization is crucial to wound healing [95]. Macrophages of different phenotypes have distinct functions in wound healing. M1-type macrophages are involved in clearing necrotic tissue and pathogens on the surface to accelerate the progression of inflammatory reactions, whereas M2-type macrophages function as anti-inflammatory cells to promote tissue remodelling and angiogenesis. The regulation of macrophage polarization and recruitment by DETCs remains controversial. According to Julie *et al.*, DETCs cannot produce hyaluronan, a glycosaminoglycan that accumulates in damaged tissues and is required for inflammatory cell motility. DETCs induce hyaluronan expression in keratinocytes by secreting KGF, which binds to the FGFR2-IIIb receptor, as mentioned above. After the induction of hyaluronic acid synthesis, macrophages migrate to wound sites [96]. Another study demonstrated that γδT cells inhibit the myeloid-mediated inflammatory response in burn wounds, including macrophage infiltration. This suggests that DETCs may facilitate the transition of the wound from an inflammatory stage to a proliferative stage of healing [97]. During wound healing, macrophages produce large amounts of nitric oxide via inducible nitric oxide synthase (iNOS), which promotes angiogenesis and facilitates the proliferative stage of wound healing. Research has indicated that the absence of γδT cells significantly decreases iNOS expression at the wound site [98]. This suggests that γδT cells play a role in the upregulation of iNOS expression during skin injury by directly influencing macrophages. Additionally, γδ T cells have been suggested to regulate the chemotaxis of macrophages to the wound site, calibrate macrophage polarization, and promote both macrophage apoptosis and pyroptosis [99]. Hu *et al.* demonstrated that Vγ4^+^ γδT cells, another γδT cell subset residing in the dermis and entering the epidermis after skin injury, can impede the phenotypic conversion of macrophages from M1 to M2 by producing IFN-γ, thereby delaying wound closure [100]. Further investigation is required to understand how DETCs interact with macrophages, including their recruitment and promotion of phenotypic transformations.

#### DETC-derived IL-17 assists in wound repair

Besides the repair mechanisms mentioned above, wound healing is influenced by IL-17, which is rapidly produced by a subset of DETCs.

Studies have shown that IL-17 receptor A (IL-17RA), is rapidly upregulated in wounded keratinocytes, making it a potential target for IL-17A signalling [101]. After DETC-mediated IL-17 signalling, epidermal host defence molecules are produced, including β-defensin 3. This molecule induces intracellular Ca^2+^ mobilization and phosphorylation of EGFR and STAT, which increases keratinocyte migration and proliferation [102]. S100 calcium binding protein A8 (S100A8) is strongly upregulated in the epidermis of acute murine and human wounds [103]. The regenerating islet-derived protein RegIIIγ activates exostosin like glycosyltransferase 3 (EXTL3)-PI3K-Akt, inducing keratinocyte proliferation but inhibiting differentiation, ultimately promoting wound healing [104]. Antimicrobial peptides and proteins not only act as defenders that kill microbes but also play a critical role in regulating keratinocyte migration, energy metabolism, cytoskeletal dynamics, and keratinocyte proliferation and differentiation [101]. MacLeod *et al.* observed the induction of antimicrobial peptides around the wound bed as early as 24 h after wounding. At 18 h after wounding, DETCs produced IL-17, confirming the antibacterial effect during the wound healing process [101]. Furthermore, a mouse model of *Staphylococcus aureus* cutaneous infection showed that DETCs are crucial for mediating neutrophil recruitment and host defence against *S. aureus* skin infections [80].

The antimicrobial functions of the skin are largely influenced by DETCs and DETC-derived IL-17. This facilitates the wound repair response, as microorganisms in the skin are detrimental to wound healing [105].

### Role of DETCs in pathologic conditions

#### Role of DETCs in UV-induced skin damage and skin tumour

The skin is highly sensitive to solar UV radiation (UVR), which can trigger DNA damage response signalling pathways after exposure. If left unchecked, this can lead to inflammation and cutaneous carcinogenesis, including basal cell carcinoma, squamous cell carcinoma, and melanoma [106].

UVR is classified into three categories based on its wavelength: UVA (315–400 nm), UVB (280–315 nm), and UVC (100–280 nm). UVA has the longest wavelength and can partially penetrate the skin, with most reaching the dermal skin. UVB mainly affects the epidermis, with only 10% penetrating the upper dermis. UVC is absorbed by the atmospheric ozone layer before it reaches the ground. UVR is a major cause of photosensitive skin diseases [107,108].

UVR can cause DNA damage by inducing chemical reactions with DNA or DNA strand breaks, helping to generate photochemical products such as cyclobutane pyrimidine dimers and pyrimidine-(6-4)-pyrimidone. This can result in genetic instability and an increased risk of cancer [109,110].

The role of DETCs in sensing solar injury and enhancing keratinocyte DNA repair response has been identified. Extracellular ATP (eATP) released from keratinocytes upon exposure to acute UVR leads to DETCs activation. This activation manifests as upregulated CD69 expression and a round phenotype. eATP directly interacts with DETCs to promote IL-17 production via purinergic P2X receptors. Furthermore, eATP binds to P2X7 on keratinocytes and induces Nod-like receptor protein 3/caspase-1-mediated IL-1 secretion. This secretion increases IL-17 production by DETCs. Enhanced IL-17 production upregulates the expression of growth arrest and DNA damage-associated gene 45 and TNF-related weak inducer of apoptosis, both of which have DNA repair functions [111]. IL-17-producing γδ T cells can enhance tumour-specific T cell responses elicited by tumour cell death [112], thereby improving the efficacy of chemotherapy. However, other studies have found that IL-17 accelerates tumorigenesis by promoting the proliferation of skin epithelial cells and formation of the tumour microenvironment [113,114]. These conflicting observations indicate that IL-17 has dual effects on skin cancer and it remains unclear whether the dual role of IL-17 is related to its concentration.

Consistent with Skint1’s normal sensing function, the Skint1–γδTCR interaction is essential in the skin’s response to UVR. This interaction enables rapid DETCs responsiveness to skin radiation via TNF receptors and induces qualitative changes that mimic the UVR response, including the expression of DETC effector genes such as *Xcl1* and *Gzmc* [39].

IL-13 may also participate in DETCs protecting against UVR-induced epidermal damage. IL-13 is expressed by DETCs in resting skin, and exposure to UV significantly upregulates its expression [21]. IL-13 has been shown to protect against cutaneous epithelial carcinogenesis induced by chemicals [21,51]. This may be associated with the finding that IL-13 promotes the maturation of upward-moving keratinocytes and controls their movement rate from the basal layer, thus regulating epithelial renewal [21]. Therefore, it is probable that DETCs enhance wound repair after UVR exposure by increasing IL-13 production, which may prevent the accumulation of mutated cells in tissues.

Given their crucial role in immune monitoring and the long-term maintenance of epidermal integrity, it is likely that DETCs use other molecules or methods to perform repair functions after UVR, such as IL-12 and nucleotide excision repair enzymes, which are involved in DNA repair and maintaining homeostasis [115]. Further studies are still needed.

DETCs exhibit specific cytolytic activity against tumour cells, as they can directly recognise, bind, and lyse skin-originating tumour cells but not normal keratinocytes *in vitro* [106,116]. Furthermore, they induce CD8+ T cell-mediated immunity against skin tumours induced by UVR [117]. However, studies have shown that the population of DETCs is reduced in the UV-irradiated epidermis [118]. The sensitivity of DETCs to UVB-induced apoptosis may partially explain how UV light induces immunosuppression. Moreover, in a UV-induced squamous cell carcinoma (SCC) model, DETCs were found to inhibit the activation of CD4+ T cells, which accelerated tumour growth, indicating their role in enabling skin tumours to evade immune-mediated destruction [119].

Although solar UVR is the primary cause of cutaneous carcinomas, they can also be induced by chemicals such as arsenic [120,121]. In models of 7,12-dimethylbenz(a)anthracene/12-O-tetradecanoylphorbol-13-acetate-induced SCC, DETCs were found to possess antitumour cytotoxicity [122]. DETCs contribute to skin cancer prevention by expressing IFN-γ and NKG2D, thereby enhancing the rapamycin-mediated therapeutic effect [122,123].

Melanoma, although comprising only 1% of all cutaneous carcinoma cases, remains highly aggressive and metastatic [124]. Studies have shown that DETCs can inhibit the growth of murine melanoma cells, both *in vitro* and *in vivo* [125]. The *in vitro* 51Cr release assay indicated that DETCs were cytotoxic to melanoma cells as well as to chemically induced cutaneous fibrosarcoma. Consistently, when injected *in vivo* with melanoma cells, DETCs significantly delayed melanoma outgrowth [125]. Furthermore, mice lacking the JAML protein were vulnerable to the formation and growth of B16F10 melanoma tumours. This suggests that the binding of JAML to CAR, which has been identified as an essential co-stimulatory mechanism for DETC activation, is crucial for the inhibitory effects of DETCs on cutaneous carcinomas [126].

**Figure 2:**
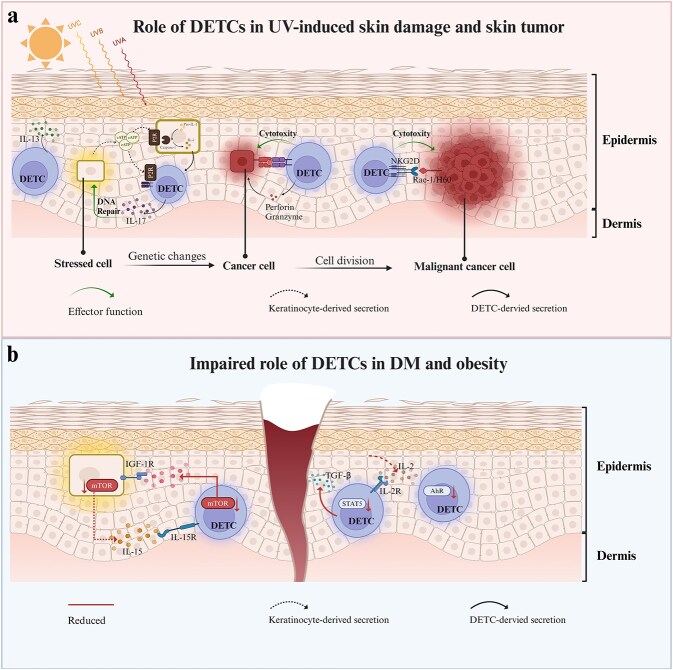
Role of DETCs in pathologic conditions. (**a**) Role of DETCs in UV-induced skin damage and skin tumours. DETCs play a role in sensing solar injury and potentiating keratinocyte DNA repair response. They also possess tumour cell-specific cytolytic abilities that directly recognise, bind, and lyse tumour cells originating in the skin. (**b**) Impaired roles of DETCs in DM and obesity. DETCs are involved in impaired wound repair in DM and obesity. The mechanisms underlying DETC dysfunction in obesity and diabetes may involve a reduction in AhR signalling in DETCs, an increase in chronic inflammation in the skin microenvironment, and interruption of cellular interactions between DETCs and keratinocytes. *DETC* dendritic epidermal T cell, *UVA* ultraviolet A, *UVB* ultraviolet B, *UVC* ultraviolet C, *IL-13* interleukin-13, *eATP* extracellular ATP, *P2R* P2 receptor, *IL-1* interleukin-1, *IL-17* interleukin-17, *NKG2D* natural killer group 2 member D, *Rae-1* retinoic acid early inducible-1, *H60* histocompatibility 60, *DM* diabetes mellitus, *IGF-1R* insulin-like growth factor 1 receptor, *IGF-1* insulin-like growth factor 1, *IL-15* interleukin-15, *IL-15R* interleukin-15 receptor, *mTOR* mammalian target of rapamycin, *TGF-β* transforming growth factor-β, *IL-2* interleukin-2, *IL-2R* interleukin-2 receptor, *STAT5* signal transducers and activators of transcription 5, *AhR* aryl hydrocarbon receptor. Created with BioRender.com

These findings suggest that DETCs, as sentinel cells in the epidermis, limit UV-induced DNA damage, play a role in repairing UVR-related injury, and even act as major antitumour agents in the murine epidermis. However, the mechanisms underlying the response of DETCs to UVR-induced skin damage, skin tumour microenvironment, and intercellular communication require further exploration to identify analogous immune signalling in the human skin or novel therapeutics (Figure 2A).

#### Impaired role of DETCs in aged skin

As the body ages, the skin thins and becomes more fragile, decreasing its wound-healing ability. Aged skin undergoes changes in multiple stages of wound healing, including prolonged inflammation, impaired immune cell function, and delayed re-epithelialization and re-vascularization, resulting in defective wound healing. These features can be contributed to by impaired cell–cell communication, increased inflammation, loss of epidermal stem cell heterogeneity, and dysregulation of stem cell potential [127,128].

Although there have been few studies on DETCs in the healing of ageing skin, existing studies have provided compelling evidence that DETCs play an important role in delayed wound repair processes in aged skin [129]. Upon skin injury in aged mice, the number of DETCs decreased compared to that in young mice. Furthermore, DETCs in aged mice were less rounded and displayed more dendrites than those in young mice. These observations suggest that keratinocytes failed to properly activate and maintain DETCs. It has been documented that epidermally expressed Skints, which are highly correlated with DETCs, are selectively upregulated at the wound edge of young mice, but not in aged mice. The IL-6/STAT3 signalling pathway in keratinocytes regulates skin expression of Skints. STAT3, which is downstream of the JAK family signalling pathways, directly regulates expression of Skints in keratinocytes. Exposure to IL-6, a canonical upstream ligand of STAT3 signalling, activates STAT3, which then enters the nucleus and regulates gene transcription [130], significantly enhancing Skint expression in epidermal keratinocytes. However, it is important to note that, whereas *Skint1* is related to the maintenance of DETCs [39], mice with knocked-down epidermal *Skint3* and *Skint9* also display delays in wound-induced re-epithelization [129].

However, IL-6 has previously been reported to be involved in cellular senescence-inducing circuits. IL-6 stimulation forms a senescence-inducing circuit involving STAT3-insulin-like growth factor-binding protein 5, which drives ROS generation. This leads to the subsequent expression of persistent and excessive DNA damage response, activating p53-pathway-dependent senescence [131]. Further investigations are required to determine whether IL-6 is a potential candidate for restoring delayed wound healing in aged skin.

### Function of DETCs in contact hypersensitivity response

Contact hypersensitivity (CHS) is an experimental murine model of human allergic contact dermatitis. It is characterized by T cell-driven skin inflammation following exposure to contact allergens [132,133]. Some studies suggest that DETCs may play a role in disease pathogenesis.

DETCs are vital for the development of contact sensitivity reactions, as shown in several studies. IL-17 amplifies the pathogenesis of CHS because allergen-specific T cell-mediated immune responses are significantly reduced in IL-17-deficient mice [134]. The number of DETCs appears to increase following exposure to 2,4-dinitrofluorobenzene in wild-type mice [135], and during CHS, activated DETCs produce IL-17 in an IL-1β-dependent manner, suggesting a role for DETCs in CHS [135]. Furthermore, allergens have been shown to increase the expression of NKG2D ligands in cultured mouse keratinocytes, such as Mult-1, H60, and Rae-1, as well as the expression of MICA in human keratinocytes. This suggests that the activation of DETCs during CHS is NKG2D-dependent [136].

Conversely, some studies have demonstrated that γδ T cells have anti-inflammatory effects on CHS [137,138]. Girardi *et al.* demonstrated that DETCs are necessary and sufficient for the downregulation of dermatitis in mice deficient in γδ T cells [139]. Therefore, DETCs may alleviate CHS responses. Furthermore, studies have found that DETCs can induce immunological tolerance, suggesting their potential role in downregulating cutaneous immune responses [140].

Besides, DETC-mediated wound healing is also attenuated by allergic skin inflammation. The overexpression of IL-4, which acts as a key component of the atopic environment [141,142], reduced the frequency and numbers of DETCs [143,144]. Wang *et al.* found that IL-4-mediated elimination of DETCs leads to a decrease in the production of prohealing cytokines including FGF-7, ultimately delaying the proper wound repair process [144]. However, the mechanism underlying the IL-4-induced loss of DETCs in the skin requires further investigation. Research on how IL-4 alters γδ T-cell populations may help us understand the interaction between DETCs and the skin inflammatory environment and identify potential therapeutic targets.

Although DETCs are involved in skin immune-mediated diseases, conflicting results regarding the role of γδ T cells in the response to contact allergens suggest that further research is still needed.

#### DETCs in impaired wound healing associated with diabetes mellitus and obesity

As the population ages, the incidence of age-related diseases such as diabetes mellitus (DM) and obesity is increasing. These diseases often lead to non-healing wounds due to diminished or altered levels of growth factors at the wound site. This impairs leukocyte infiltration, cell growth, and migration in wounds [145,146]. A comprehensive understanding of these changes and their interrelationships may aid in developing targeted treatments and improving patients’ quality of life.

Although most studies have concentrated on dermal cells such as macrophages and neutrophils, DETCs located in the epidermis also participate in impaired wound healing in DM and obesity. Dysfunction of DETCs in obesity and diabetes, two of the most common metabolic diseases, is complicated and not yet fully understood (Figure 2B).

One factor that may play a role in this process is the aryl hydrocarbon receptor (AhR), a ligand-activated cytoplasmic transcription factor. AhR signalling is widely distributed in various tissues and has been identified as a potential regulator of glucose balance and lipid metabolism [147]. AhR plays an essential role in the immune system by regulating inflammation and cellular metabolism. In the epidermis, AhR directly mediates transcription of c-Kit, which is involved in DETC homeostasis. AhR-deficient mice exhibit an inability of DETCs to adopt dendritic morphology and proliferate sufficiently after seeding the skin, ultimately causing the loss of DETCs shortly after birth [24]. In the absence of AhR, the inflammatory tone of DETCs increased. Genes related to the cluster-group ‘inflammation’, including *IFN-γ, granzyme F*, and *programmed death-ligand 1*, were upregulated in Ahr^−/−^ mice. This suggests a dampening role of AhR in the inflammatory profile of DETCs in healthy skin. Furthermore, AhR signalling also participates in proper activation and morphology of DETCs, since such signalling is necessary for genes involved in actin metabolism and ion homeostasis, such as Fermt2 and Kcnma1, which encode a subunit of calcium-regulated large conductance (BK) potassium channels. Experimental studies have revealed that AhR signalling is reduced in obesity [148]. Therefore, it is reasonable to speculate that obesity induces a reduction in AhR signalling, which further prevents DETCs from rounding up and releasing cytokines upon activation. This ultimately results in delayed healing of the wound.

Chronic inflammation contributes to DETC dysfunction in obesity and diabetes. IL-2 stimulates DETC proliferation by binding to the IL-2 receptor, activating Jak1 and Jak3, resulting in STAT5 phosphorylation and translocation of the STAT5 complex to the nucleus, where it regulates gene transcription [149,150]. Hyperglycaemic conditions have been found to alter IL-2 and STAT5 signalling, resulting in a 50% reduction in skin γδ T cells in the epidermis. The remaining epidermal γδT cells cannot perform tissue repair functions and are unable to upregulate TGF-β production at the wound edge [151]. These defects contribute to an increase in chronic inflammatory molecules including TNF-α, as in obese mice blocking TNF-α has been shown to restore DETC function during injury repair [151].

Disruption of the IGF-1–mTOR–IL-15 loop, which leads to the interruption of cellular interactions between DETCs and keratinocytes, also contributes to DETC dysfunction in obesity and diabetes.

mTOR, a highly conserved kinase belonging to the PI3K family [152], is involved in this process. There are two distinct complexes, mTOR complex 1 (mTORC1) and mTORC2. These complexes exhibit different features and functions. mTORC1 regulates cellular growth and metabolism, and is sensitive to rapamycin. In contrast, mTORC2 regulates cell survival and cytoskeletal remodelling but is not sensitive to rapamycin [152,153].

Research has suggested that IGF-1, which is derived from DETCs, activates mTOR signalling in keratinocytes. This stimulates keratinocytes to produce IL-15. Furthermore, IL-15 mediates IGF-1 production by DETCs, in which mTOR also participates [154]. When mTOR activation was inhibited by rapamycin, IGF-1 secretion decreased, resulting in reduced IL-15 production. This ultimately presented as disturbed DETC homeostasis and impaired wound healing [74,154]. Therefore, IGF-1 derived from DETCs and IL-15 derived from keratinocytes contribute to the production of each other, constituting a regulatory feedback loop in which mTOR acts as a key mediator. The mTOR pathway plays a pivotal role in the pathogenesis of diabetes by regulating the energy balance and metabolism. Weak activation of the mTOR pathway has been observed in the intact skin of streptozotocin-induced diabetic rats [155]. In diabetic mice, mTOR activation is suppressed in the epidermis, disrupting the IGF-1–mTOR–IL-15 loop. This results in the attenuated wound repair function of DETCs and ultimately delays wound healing [154].

In humans, the number of γδ T cells in peripheral blood is negatively correlated with the severity of obesity. γδT cells from obese donors also exhibit reduced levels of IL-2Rα, the ligand of which can restore γδT cell antiviral cytokine production, indicating impaired homeostasis and antiviral function of γδT cells in peripheral blood due to obesity [156]. Epidermal γδT cells isolated from human chronic wounds are resistant to further stimulation and present an unresponsive state. Studies have shown that IGF-1 levels decrease in non-healing wounds of patients with diabetes [10,157]. These experimental results suggest that epidermal γδT cells in both mice and humans are affected by the pathological status of diabetes and obesity and participate in disordered wound healing. Since chronic inflammation, central to refractory diabetic wound healing, is strongly related to inflammaging and can result in immune exhaustion similar to that observed in HIV infection or cancer [158], future research should investigate whether exhaustion, anergy, or senescence of DETCs exists in such refractory wounds and how these pathological states contribute to impaired wound repair.

### Current technical advances in DETCs research

To conduct further research on DETCs, mastering specific DETC-related experimental approaches is essential. Several protocols are available [159,160], with core technologies mainly focusing on the isolation and culture of DETCs as well as assessing DETC activation *in vivo* and *in vitro*.

The preparation of an epidermal single-cell suspension is usually accomplished using a two-step digestion method. First, the epidermis is dissociated from the dermis; then, the epidermis is digested to create a single-cell suspension. Methods involved include enzymes, heat, and mechanical digestion. Trypsin solution is most commonly used, with collagenase and dispase as alternatives [151,154]. Isolation of DETCs from the epidermal single-cell suspension can be achieved using magnetic bead separation [22], fluorescence-activated cell sorting [151], or density gradient separation [93]. Ibusuki *et al.* sorted DETCs with magnetic beads using a two-step method [22]. They first stained cells with a phycoerythrin (PE)-conjugated anti-integrin β7 monoclonal antibody (mAb), exploiting the fact that DETCs are the only cells in the normal epidermis expressing the integrin β7 chain. They then incubated the cell suspension with anti-PE mAb-conjugated magnetic particles conjugated so that the labelled cells could be well isolated.

For flow cytometry, different gating strategies can be applied. For example, epidermal cells could be gated on live Thy1.2+ and γδ TCR+ to distinguish γδ T cells [151]. Alternatively, DETCs can be sorted by fluorescence-activated cell sorting based on Thy1.2 and Vγ3 expression [101]. Otherwise, DETCs were sorted as live CD45 + CD3 + TCRδ+ [39]. The harvested cells can be then be analysed using techniques such as flow cytometry and RNA-seq to investigate the effects of various factors on DETCs and downstream signalling pathways.

Regarding the culture of DETCs, it is worth noting that, in addition to the regular cell culture medium containing fetal bovine serum (FBS), cytokines such as IL-2 and IL-15 and intermittent TCR stimulation are also required to maintain T-cell proliferation and survival in culture [161].

DETCs maintain their characteristic dendritic morphology at a steady state, retracting their dendrites and taking on a rounded morphology upon activation. Therefore, by observing these morphological changes, we can gain insights into DETC function under both physiological and pathological conditions. Harvested back skin tissue is first embedded in optimal cutting temperature (OCT) compound, cryosectioned to a thickness of ≥10 μm, and then subjected to specific immunofluorescence staining [162]. Alternatively, whole depilated split ear sheets can also be fixed, permeabilised, blocked, and then immunofluorescently stained [39]. Usually, DETCs are visualized using fluorescent reagents conjugated to Vγ3, γδTCR, or CD3, all of which are molecular markers of DETCs.

*Ex vivo* organ culture is another important research method. This technique provides an opportunity to better understand complex biology in a physiologically relevant context. Typically, murine ears are split and allowed to float with epidermis side-up in a culture medium [76]. The epidermal sheets can then be treated under stimulatory conditions according to different experimental requirements prior to subsequent analyses [39,111].

Intravital labelling with intravital dynamics microscopy is another method that can better visualize the morphology and state of DETCs *in vivo*. After anaesthesia, the ear pinnae of the mice are injected with labelling solutions, typically containing fluorescent reagents such as a fluorescently labelled anti-Vγ5 antibody [14]. Alternatively, green fluorescent protein (GFP) reporter mice can also be used [15]. The mice are placed on a heated microscope stage with their ear pinna immobilized on a metal pedestal, which is then moistened with a drop of phosphate buffered saline (PBS) and covered with a glass coverslip before imaging. Furthermore, to investigate the dynamics of granules inside DETCs, Chodaczek *et al.* marked the granules via intradermal injection of a fluorescent dye conjugated with a TCR antibody [15]; this method enabled the visualization of intracellular vesicles and the study of *in vivo* trafficking of intracellular cargo within DETCs using intravital microscopy.

## Conclusions

DETCs, acting as sentinels, are crucial components in the epidermis both in quantitative and functional terms. They play roles in immune surveillance, maintaining skin homoeostasis, and promoting wound healing and tissue regeneration. Owing to the similarity between human skin γδT cells and mouse DETCs, further research on DETCs can provide valuable insights into the role of T cells in human skin. Under various pathological conditions, such as ageing, UV radiation, DM, and obesity, the normal physiological functions of DETCs are impaired. Concurrently, DETCs also alternatively contribute to these pathological conditions by disturbing normal cell–cell or cell-molecular interactions. Therefore, it is crucial to investigate how DETCs and the skin microenvironment interact in further explorations.
